# Absolute Configuration Assignment From a Single Reflection Using Resonant X‐Ray Diffraction

**DOI:** 10.1002/chir.70129

**Published:** 2026-08-04

**Authors:** Anmol Andotra, Fabian Gasser, Ferdinando Malagreca, Roland C. Fischer, Yves H. Geerts, Roland Resel

**Affiliations:** ^1^ Institute of Solid‐State Physics Graz University of Technology Graz Austria; ^2^ Université Libre de Bruxelles (ULB), Faculté des Sciences, Laboratoire de Chimie des Polymères Bruxelles Belgium; ^3^ Institute of Inorganic Chemistry Graz University of Technology Graz Austria; ^4^ International Solvay Institutes of Physics and Chemistry Université Libre de Bruxelles (ULB) Bruxelles Belgium

**Keywords:** absolute configuration, chirality, organic single crystals, resonant scattering

## Abstract

Determination of the absolute configuration of chiral molecules is essential across multiple scientific disciplines, as handedness determines the chiroptical, biological, and pharmaceutical properties. For crystalline materials, single crystal x‐ray diffraction is the standard technique for assigning the absolute configuration. However, its application typically requires a large number of reflections and analysis of Friedel pair intensities using the Flack parameter or related approaches, such as those of Hooft or Parsons. Here, we introduce a conceptually distinct approach for determining the absolute configuration of a known crystal structure, based on the x‐ray energy dependent intensity of a single Bragg reflection that is sensitive to the absolute configuration. By monitoring the variation in the intensity of a Bragg peak across an absorption edge, this method provides an unambiguous probe of chirality. The proposed approach is demonstrated for the 211 reflection of a 2‐(3‐bromophenyl)‐*N,N*‐diisopropyl‐2‐oxoacetamide single crystal, showing that the absolute configuration can be reliably determined from a single reflection. To confirm the assignment of chirality and assess potential limitations, the x‐ray energy dependence of additional diffraction peaks was also investigated. This method is particularly advantageous for systems with a limited number of accessible reflections, where conventional statistical methods based on Flack parameter analysis are not reliable.

## Graphical abstract

A method to determine the absolute configuration of chiral crystals is introduced. It takes advantage of the intensity of a single x‐ray diffraction peak that is sensitive to the absolute configuration as a function of energy.

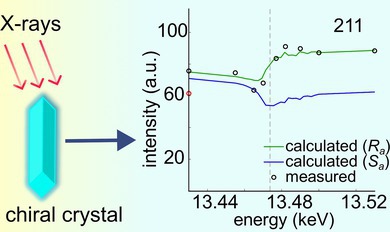

## 1 Introduction

An object that cannot be superimposed on its mirror image is defined as chiral. Chirality is of particular importance because it directly influences chemical reactivity, biological function, and a wide range of physical properties [1, 2]. Consequently, the reliable determination of absolute configuration in crystalline systems is essential for enabling applications across various field [3]. A variety of direct and indirect methods have been developed to determine absolute configuration [4, 5, 6]. Among these, single crystal x‐ray diffraction (SCXRD) remains one of the most widely used and powerful techniques. Its sensitivity to chirality was first recognized by Coster and Knol in 1930 [7] and subsequently demonstrated for organic compounds by Bijvoet in 1949 [8] through resonant (anomalous) scattering effects and their influence on peak intensities. In SCXRD, the absolute configuration is conventionally determined using the Flack parameter [9] which is obtained by refining the relative contributions of a structure and its inversion twin. It is evaluated by exploiting intensity differences between Friedel pairs (hkl and $\bar{h}\bar{k}\bar{l}$) that arise from resonant scattering. In recent years, this approach has been complemented and refined by additional statistical models, such as the Hooft [10, 11, 12] and Parsons methods [13, 14]. Due to its accessibility, and continuous methodological and instrumental advancements over recent decades, SCXRD has become the benchmark technique for absolute configuration determination [15].

Beyond conventional SCXRD, synchrotron‐based resonant diffraction techniques provide additional opportunities to exploit x‐ray energy dependent scattering phenomena. Diffraction anomalous fine structure (DAFS) spectroscopy is one such technique, belonging to the broader class of resonant x‐ray diffraction methods [16, 17]. In DAFS, the x‐ray energy dependence of a Bragg reflection is analyzed, allowing to probe local structural information, such as short‐range order around a specific atomic species in mixed amorphous and nanocrystalline systems, or to investigate composition, strain, and electronic structure in semiconductor materials [18, 19].

Here, we introduce a conceptually distinct approach to assign the absolute configuration of a known crystal structure. The method exploits the x‐ray energy dependent intensity of a single Bragg reflection that is sensitive to the absolute configuration, enabling direct assignment. By monitoring the variation in peak intensities across an absorption edge, this method provides an unambiguous probe of chirality. In contrast to conventional SCXRD, which typically relies on a statistically significant number of Friedel pairs, our approach is well suited to systems with a limited number of accessible reflections and does not require reliance on conventional Flack parameter analysis.

## 2 Theoretical Background

From a crystallographic standpoint, identification of the space group represents a crucial first step in assessing chirality. Chiral molecules crystallize exclusively in one of the 65 Sohncke space groups [20], which contain only symmetry operations of the first kind (rotations and translations) and lack inversion or mirror symmetry. In chiral crystals, this absence of improper symmetry allows the distinction between a structure and its inverted image through differences in the intensities of Friedel pairs, hkl and $\bar{h}\bar{k}\bar{l}$. These intensity differences originate from resonant (anomalous) scattering, which arises from x‐ray energy dependent variations of the atomic scattering factor f near an absorption edge where [21, 22],

(1) $$f\left(q,E\right)=f_{0}\left(q\right)+f^{\prime }\left(E\right)+if^{\prime \prime }\left(E\right)$$

The term $f_{0}\left(q\right)$ describes the contribution originating from the Thomson scattering factor, which only depends on the length of the scattering vector q while $f^{\prime }\left(E\right)$ and $f^{\prime \prime }\left(E\right)$ are x‐ray energy dependent components associated with resonant scattering.

The x‐ray energy dependence of the intensity of a given reflection hkl arises from the x‐ray energy dependence of the structure factor. The structure factor for a reflection hkl is given by [22],

(2) $$F_{\mathrm{hkl}}\left(E\right)=\sum_{j}f_{j}\left(q_{\mathrm{hkl}},E\right)\exp\left[2\mathrm{\pi i}\left(hx_{j}+ky_{j}+lz_{j}\right)\right]\exp\left[-B_{j}\frac{q_{2}^{\mathrm{hkl}}}{{\left(4\pi \right)}^{2}}\right]$$

where $f_{j}$ is the atomic scattering factor of an atom j and $x_{j},y_{j},\text{and}\;z_{j}$ are its fractional co‐ordinates in the unit cell. $q_{\mathrm{hkl}}$ is the length of the scattering vector for the Bragg peak hkl and the last term in the equation is the Debye–Waller factor, where $B_{j}$ is the isotropic displacement parameter [23].

In practice, the experimentally measured diffracted intensities are affected by several instrumental and sample‐dependent factors. Consequently, appropriate correction factors must be applied to account for these influences enabling to extract reliable structural information. The relationship between the measured intensities and the structure factors $\left(F_{\mathrm{hkl}}\right)$ can therefore be expressed through a set of standard intensity correction terms as [22, 24]

(3) $$I_{\mathrm{hkl}}\propto I_{0}\cdot P\cdot L\cdot \lambda^{3}\cdot T\cdot {\left|F_{\mathrm{hkl}}\right|}^{2}$$

where $I_{0}$ is the intensity of the incident x‐ray beam, P and L denote the polarization and Lorentz correction factors, respectively, λ is the x‐ray wavelength, and T is the transmission coefficient accounting for absorption within the sample.

Among these factors, the polarization and Lorentz terms can be treated as x‐ray energy independent and thus play only a minor role in the analysis of x‐ray energy dependent intensity variations. The x‐ray energy dependence of $I_{0}$ and λ can be accurately monitored and readily corrected. In contrast, the transmission coefficient T, which reflects the x‐ray energy dependent absorption behavior of the sample, is particularly critical for measurements performed near an absorption edge of one of the constituent elements.

The transmission coefficient can be described using the Beer–Lambert law [25, 26],

(4) $$T\left(E\right)=e^{-\mu \left(E\right)t}$$

where μ is the linear attenuation coefficient of the sample and t is the effective path length of the X‐rays through the sample for a given reflection. The linear attenuation coefficient is directly related to the imaginary (absorptive) component of the atomic scattering factor [22] and can be expressed as

(5) $$\mu \left(E\right)=2\rho r_{e}N_{A}\frac{\mathrm{hc}}{E}\sum_{i}g_{i}f_{\prime \prime }^{i}\left(E\right)$$

where ρ is the mass density of the absorbing medium, $r_{e}$ is the classical electron radius, $N_{A}$ is Avogadro's constant, h is Planck's constant, c is the speed of light, E is the x‐ray energy, $g_{i}$ is the mass fraction of element i in the absorbing material, and $f_{i}^{\prime \prime }$ is the corresponding imaginary component of the atomic scattering factor.

## 3 Experimental

### 3.1 Sample Preparation

**FIGURE 1:**
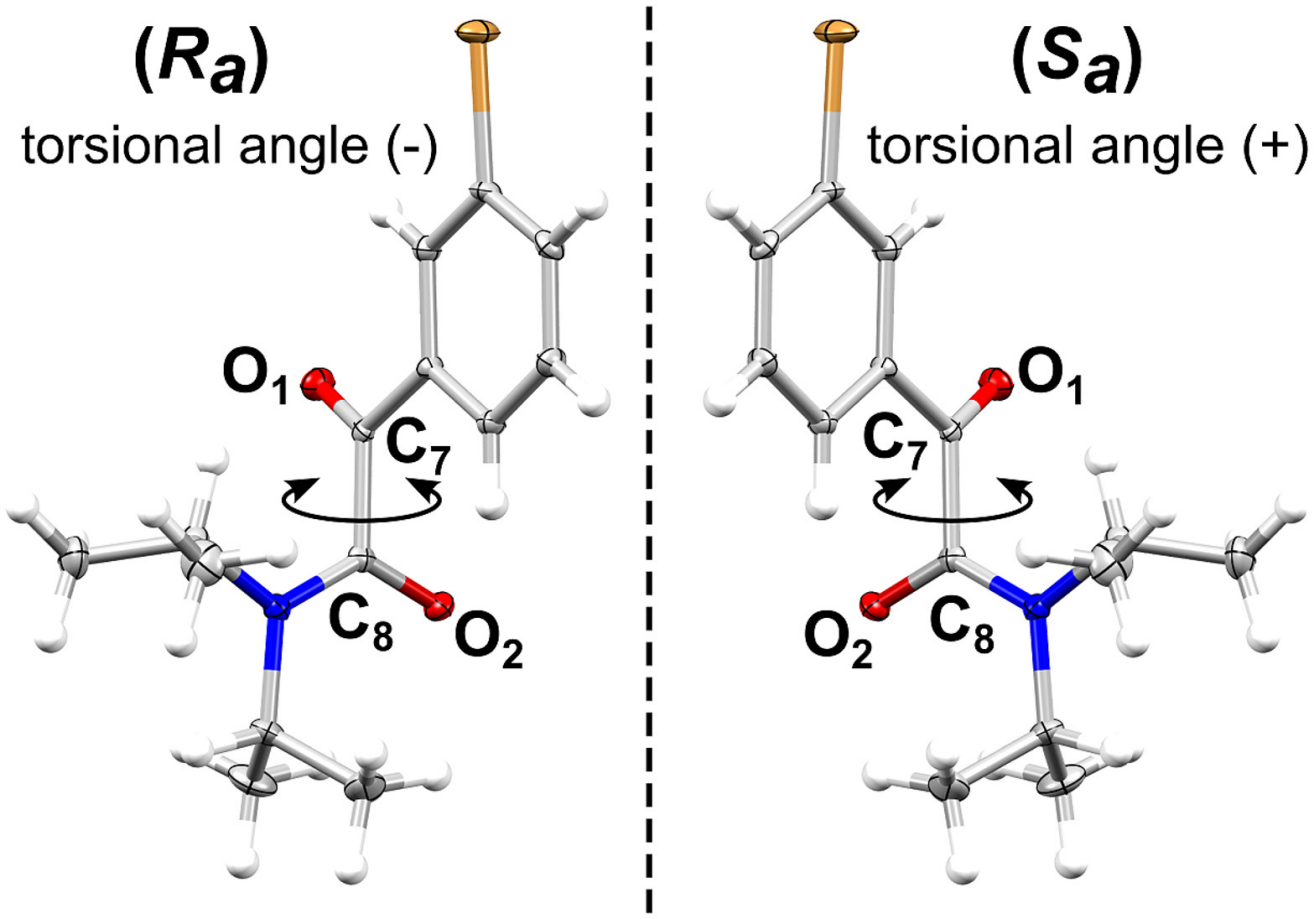
Crystal structure of the Br‐oxo shown in an ORTEP view with thermal ellipsoids at 50% probability for both the $R_{a}$ and $S_{a}$ enantiomers. A negative torsional angle about the O_1_=C_7_–C_8_ = O_2_ axis give rise to $R_{a}$ enantiomer, while a positive angle corresponds to the $S_{a}$ enantiomer.

2‐(3‐Bromophenyl)‐*N,N*‐diisopropyl‐2‐oxoacetamide (Br‐oxo) was synthesized according to established literature procedures and purified by sublimation [27]. Single crystals of the Br‐oxo suitable for x‐ray diffraction were obtained by slow evaporation of a saturated methanol solution (15 mg/mL) at ambient temperature. Upon crystallization, the Br‐oxo forms enantiomorphic crystals corresponding to two possible absolute configurations, $R_{a}$ or $S_{a}$ [27]. These configurations arise from conformational chirality associated with negative or positive torsional angle, respectively, about the O_1_ = C_7_–C_8_ = O_2_ axis (Figure 1). The descriptors $R_{a}$ and $S_{a}$ denote right‐ and left‐handedness, respectively, following the Cahn–Ingold–Prelog (CIP) convention [28].

### 3.2 Resonant X‐Ray Scattering

**FIGURE 2:**
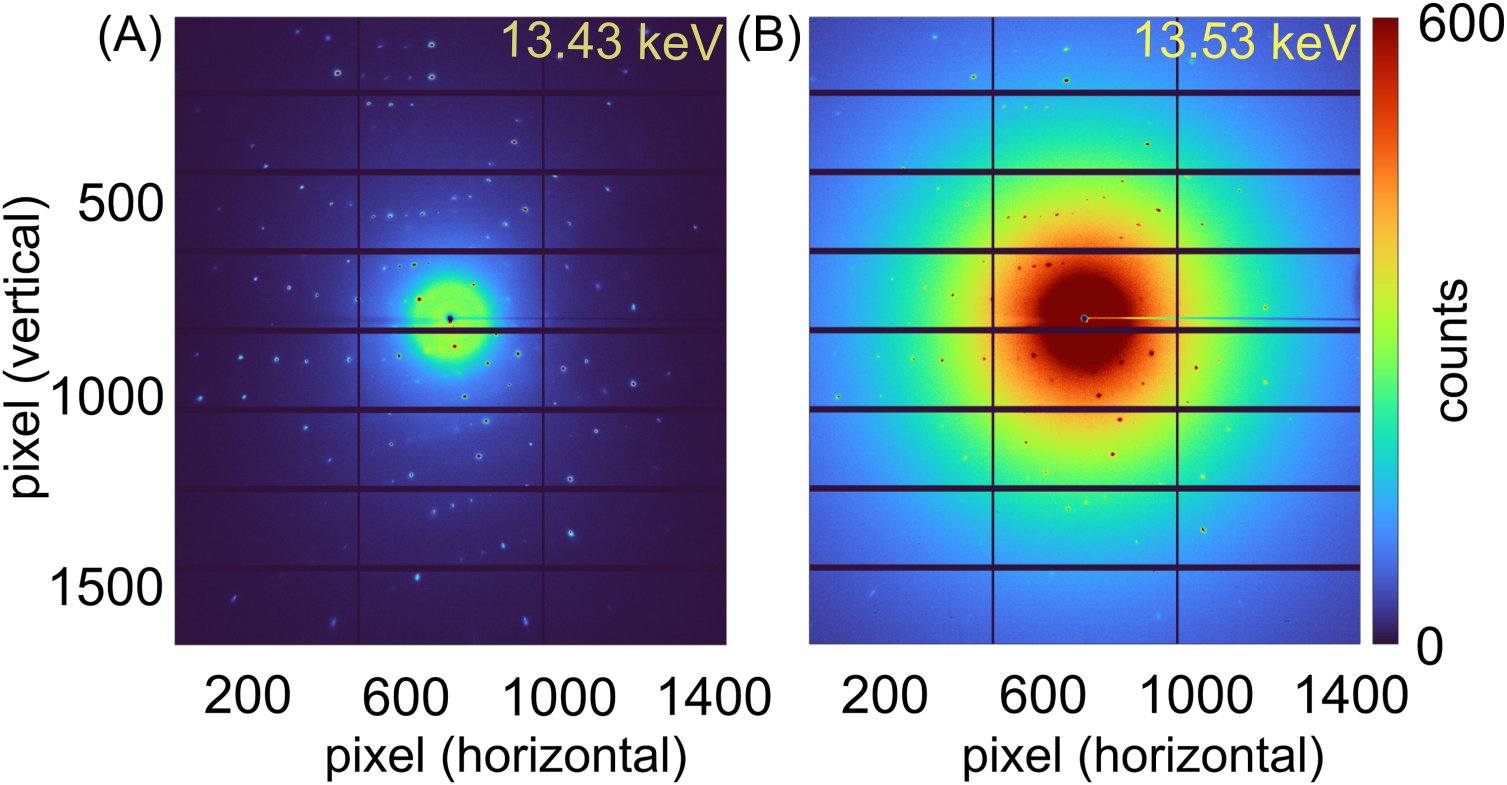
Illustrative diffraction frames of the Br‐oxo single crystals from single crystal x‐ray diffraction collected at (A) 13.43 keV (below) and (B) 13.53 keV (above) the Br K‐edge.

Resonant scattering and fluorescence experiments across the bromine K‐edge were performed at beamline XRD1, Elettra Synchrotron (Trieste, Italy). The Br‐oxo crystal with an approximate thickness of 90 μm was picked up using a small amount of NVH oil and mounted on a nylon loop attached to the goniometer head. Diffraction data were collected at 100 K using a nitrogen Cryostream 700, and at nine nominal x‐ray energies ranging from 13.43 to 13.53 keV. For each selected x‐ray energy, a total of 720 diffraction frames were collected with an exposure time of 0.2 s per frame using a Dectris Pilatus2M area detector positioned at a fixed distance of 0.086 m from the sample. Two representative diffraction patterns collected at 13.43 keV (below the absorption edge) and 13.53 keV (above the absorption edge) are shown in Figure 2. Comparison of the diffraction patterns (below and above the absorption edge) shows a notable decrease in peak intensity when measuring above the edge at 13.474 keV (Figure S1).

### 3.3 Extraction of Resonant Contributions to the Atomic Scattering Factor

Experimental determination of the x‐ray energy dependence of the atomic scattering factor f is essential for reliable interpretation of the resonant diffraction measurements. The imaginary component $f^{\prime \prime }\left(E\right)$ is directly related to the refractive index and therefore can be determined experimentally from absorption measurements. Subsequently, $f^{\prime }\left(E\right)$ can be obtained using the Kramers–Kronig transformation [29].

**FIGURE 3:**
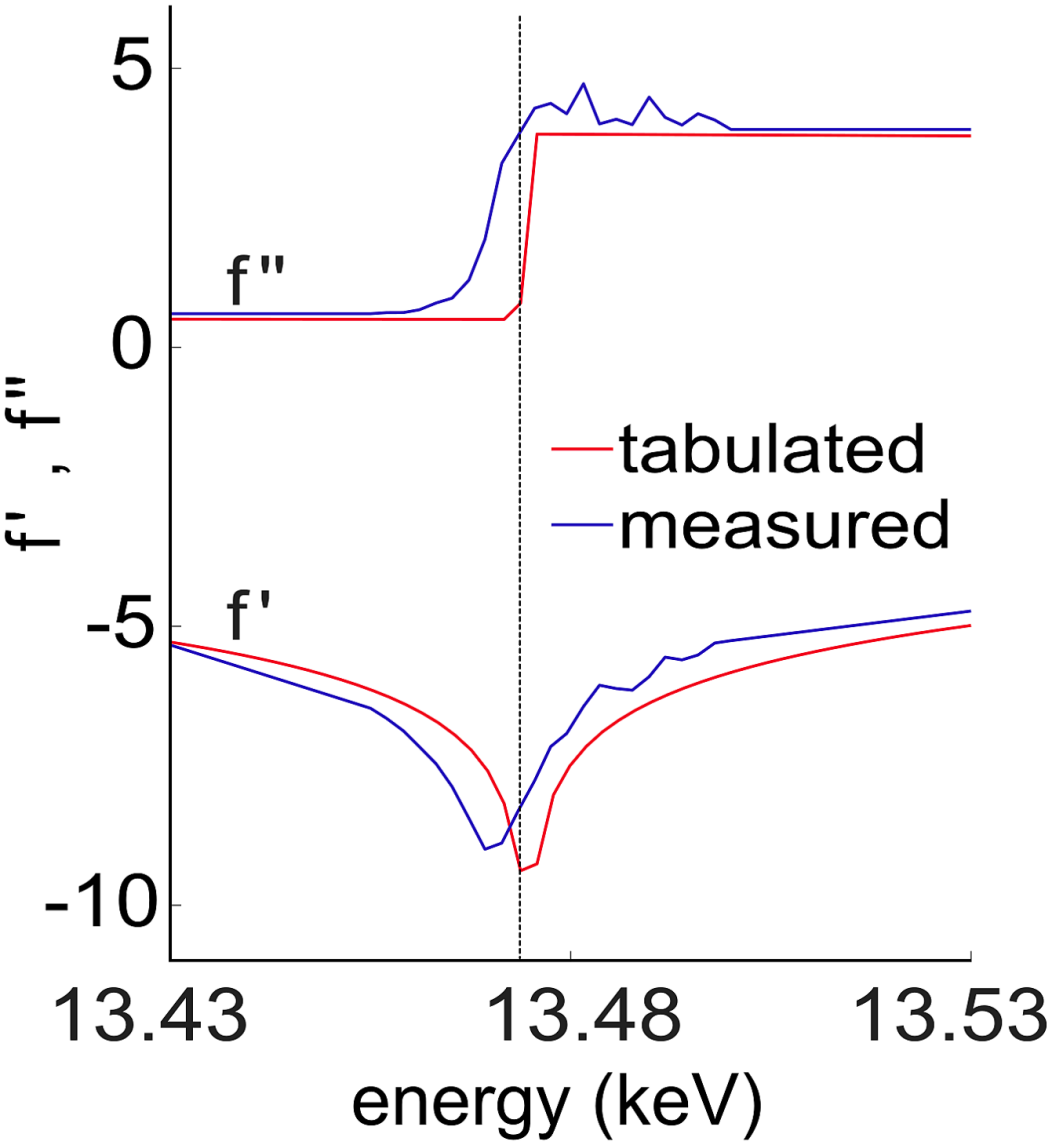
Comparison of the dispersion corrections $f^{\prime }$ and $f^{\prime \prime }$ of tabulated free‐atom Br values (red) with dispersion corrections obtained from the Br‐oxo single crystal fluorescence data evaluated using the software KKcalc (blue) across the Br K‐edge.

In this work, the $f^{\prime \prime }$ values were obtained from the x‐ray fluorescence signal emitted from the crystal and recorded using an Amptek X‐123 silicon drift detector during variation of the incident photon x‐ray energy across the bromine K‐edge. An increase in the measured background intensity was observed at energies above the bromine K‐edge due to fluorescence emission from the crystal, as shown in Figure 2B and Figure S1. From the experimentally determined x‐ray fluorescence values between 13.43 and 13.53 keV, $f^{\prime }$ and $f^{\prime \prime }$ profiles were calculated using KKcalc software [30] as shown by the blue curve (Figure 3). In comparison with tabulated values for an isolated bromine atom [31, 32] (red curve in Figure 3), the measured spectra show a slight shift of the Br K‐edge to lower energies. This shift may arise from some experimental inaccuracies or reflect the influence of the chemical environment of bromine within the Br‐oxo crystal due to its electronegative nature. Organic bromine compounds have been reported to exhibit red shifted absorption edge energies, as observed for the Br‐oxo crystal [33]. Above the absorption edge, the data exhibit some intensity fluctuations, which may indicate the presence of extended oscillations, as commonly observed for bromine containing organic compounds in x‐ray absorption spectroscopy [29, 33].

## 4 Results

Diffraction patterns recorded at 13.43 keV were first analyzed to extract peak positions, which were transformed into reciprocal‐space coordinates and indexed using the known orthorhombic crystal structure with one molecule in the asymmetric unit (Table S1).

**FIGURE 4:**
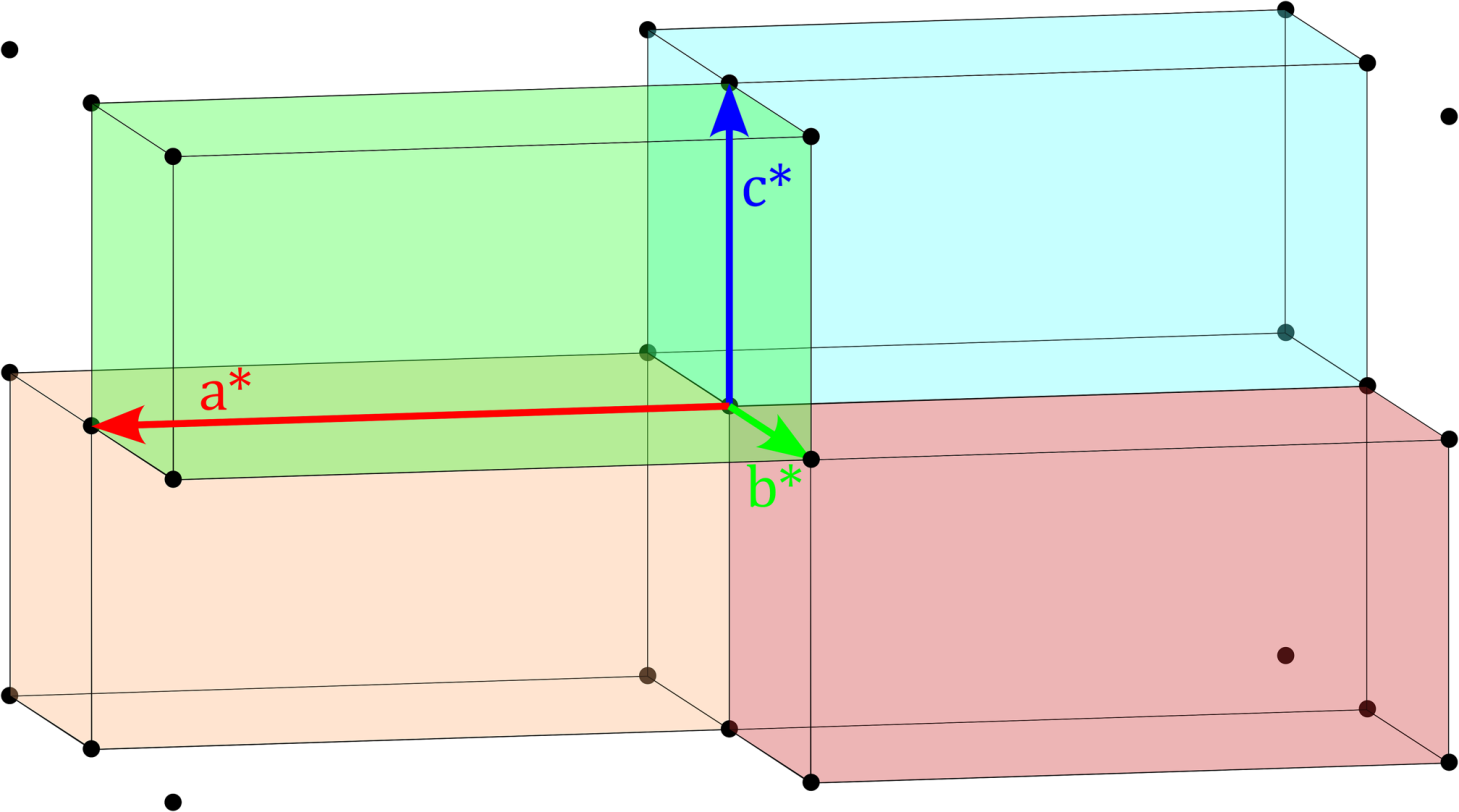
Schematic representation of the four equivalent crystallographic orientations describing the same reciprocal lattice of the investigated orthorhombic unit cell of the Br‐oxo single crystal. The four reciprocal unit cells each represent one possible crystallographic orientation and are related by $180^{{}^{\circ}}$ rotations about the reciprocal unit cell axes $a^{*}$, $b^{*}$, and $c^{*}$.

However, owing to the symmetry of the peak positions of the investigated orthorhombic Br‐oxo single crystal, no unique orientation could be identified. Instead, four crystallographic orientations were found to equally describe the measured reciprocal lattice points. As illustrated schematically in Figure 4, the four possible orientations, visualized by the green, blue, red, and orange reciprocal unit cells, are related by $180^{{}^{\circ}}$ rotations about the reciprocal unit cell axes $a^{*}$, $b^{*}$, and $c^{*}$. Consequently, a unique peak indexing is not possible, but instead any measured reciprocal lattice point can be assigned to four related Laue indices: hkl, $h\bar{k}\bar{l}$, $\bar{h}\bar{k}l$, and $\bar{h}k\bar{l}$. The measured Br‐oxo single crystal belongs to the space group *P*2_1_2_1_2_1_, for which all four index assignments represent symmetry equivalent reflections. Consequently, any of the four possible orientations leads to identical peak positions and intensities, including with respect to their resonant scattering behavior. Therefore, one of the four crystallographic orientations was arbitrarily fixed and used throughout this work.

For the assignment of the absolute configuration, the x‐ray energy dependence of the structure factor was calculated based on the experimentally determined atomic form factor of bromine atom (following Equation 2) in combination with the reported crystal structure of the Br‐oxo [27]. Analysis of the squared modulus of the structure factors reveals that the Friedel pair 211, and $\bar{2}\bar{1}\bar{1}$ exhibit markedly different x‐ray energy dependences for the two absolute configurations ($R_{a}$ and $S_{a}$), highlighting their sensitivity to the chirality of the Br‐oxo crystal. An overview of the sensitivity of different Friedel pairs to the absolute configuration of the Br‐oxo crystal is provided in Figure S2.

**FIGURE 5:**
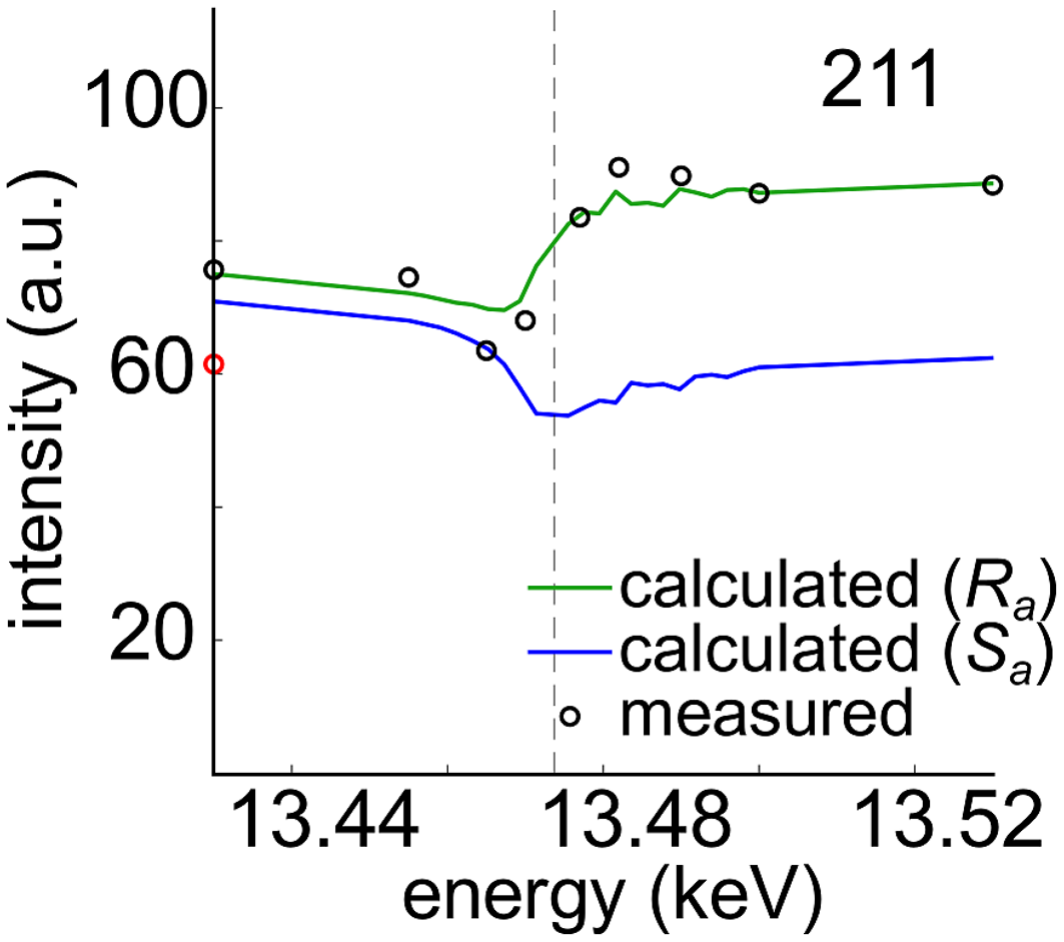
Comparison of measured 211 Bragg peak as a function of x‐ray energy with calculated structure factors for the (*R*_*a*_) and (*S*_*a*_) absolute configurations of the Br‐oxo. The red marker highlights an additional measurement collected at the starting x‐ray energy of 13.43 keV after completion of the x‐ray energy dependent measurement series.

On the basis of these results the intensity of the 211 reflection was extracted from the data collected at x‐ray energies ranging from 13.43 to 13.53 keV. The experimentally observed intensity for the 211 peak increases across the bromine K‐edge. Comparison of the experimentally observed intensity variation with the calculated x‐ray energy dependent structure factors unambiguously identifies the $R_{a}$ absolute configuration as the correct enantiomer (Figure 5).

**FIGURE 6:**
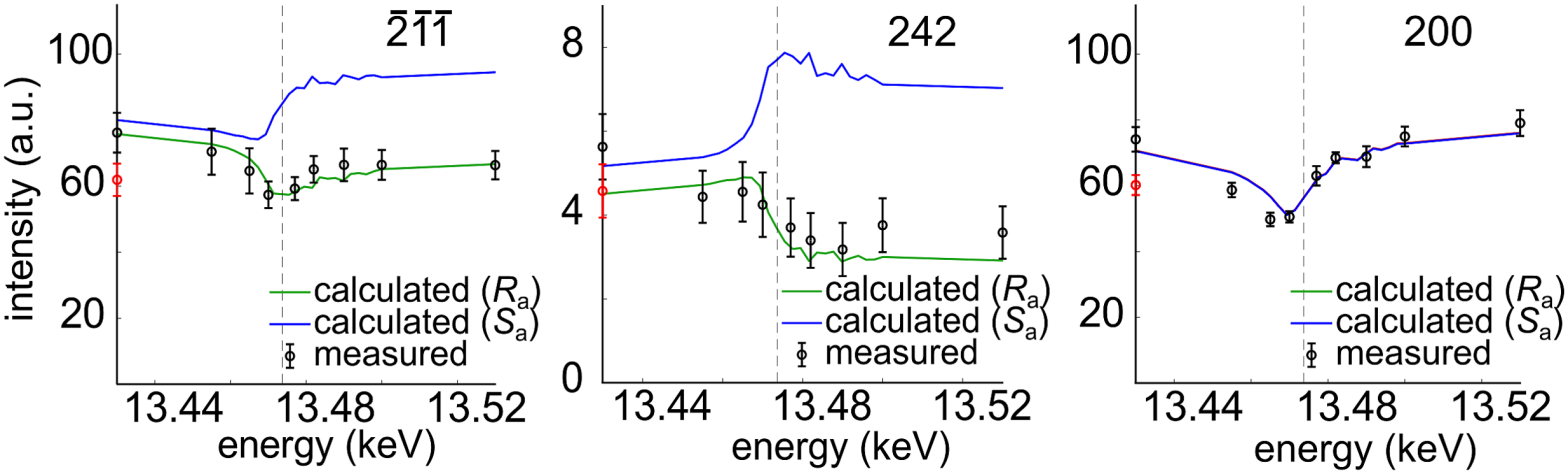
Comparison of measured $\bar{2}\bar{1}\bar{1}$, 242, and 200 Bragg peaks as a function of x‐ray energy with calculated structure factors for the (*R*_*a*_) and (*S*_*a*_) absolute configurations of the Br‐oxo. The x‐ray energy dependence of the calculated intensities of $\bar{2}\bar{1}\bar{1}$, 242, and 200 reflections was obtained by averaging over all symmetry‐equivalent reflections, allowing error bars to be determined. The red markers highlight measurements collected at the starting x‐ray energy of 13.43 keV after completion of the x‐ray energy dependent measurement series.

To further confirm the assignment, the x‐ray energy dependent intensity of the $\bar{2}\bar{1}\bar{1}$ reflection was extracted from the measured data and compared with the calculated x‐ray energy dependent structure factors. For the $\bar{2}\bar{1}\bar{1}$ reflection, the measured intensity decreases and exhibits a minimum at the bromine K‐edge (Figure 6), supporting the assignment of the $R_{a}$ absolute configuration. The error bars shown in Figure 6 arise from averaging over the symmetry‐equivalent reflections at the different energies. A similar assignment is obtained for the averaged intensities of the 211 reflection, with error bars as shown in Figure S3.

Another Friedel pair 242, and $\bar{2}\bar{4}\bar{2}$ was also analyzed. Although these reflections have relatively small structure factors, they show a large relative intensity difference between the Friedel pair (see Figure S2). The measured intensity for 242 reflection clearly decreases across the edge, further confirming the $R_{a}$ absolute configuration (Figure 6), whereas the $\bar{2}\bar{4}\bar{2}$ reflection shows opposite behavior, as expected (Figure S3). Due to their low structure factors, the error bars arising from averaged symmetry equivalent reflections are slightly larger for the 242 and $\bar{2}\bar{4}\bar{2}$ reflections. Nevertheless, the observed x‐ray energy dependence remains in good agreement with the calculated.

In contrast, calculations show that the Friedel pair 200 and $\bar{2}00$ have identical x‐ray energy dependent structure factors for both enantiomers. The measured intensity of the averaged 200 reflection, including its symmetry‐equivalent $\bar{2}00$ reflection, follows the calculated behavior and therefore carries no information on the absolute configuration (Figure 6).

Finally, the resulting assignment of the absolute configuration of Br‐oxo was also confirmed by conventional single‐crystal x‐ray diffraction (Section S2). The Flack parameter (Table S1) confirms the correctness of the assignment, showing that the Br‐oxo crystal has the $R_{a}$ absolute configuration.

To assess the impact of x‐ray exposure, a second data set was collected at the starting x‐ray energy (13.43 keV) after completion of the x‐ray energy dependent scan. The comparison of the intensities of the 211, $\bar{2}\bar{1}\bar{1}$, 242, and 200 peaks reveal an intensity reduction of approximately 10%–20% for each peak after a total illumination time of 20 min at 100 K, as highlighted by the red markers in Figure 5 and Figure 6. Although moderate radiation damage is observed, it does not compromise the assignment of the absolute configuration (Section S2).

## 5 Discussion

The absolute configuration of the Br‐oxo crystal was determined to be *R*_*a*_ by monitoring the intensity of a single Bragg reflection 211, while scanning the incident x‐ray energy across the bromine K‐edge at nine discrete energies. The measured intensity variation of the $\bar{2}\bar{1}\bar{1}$, 242, and $\bar{2}\bar{4}\bar{2}$ reflections agree with the calculated x‐ray energy dependent structure factors, further confirms this assignment.

The Br‐oxo molecule contains a heavy bromine atom; however, similar experiments can be performed for materials composed of lighter atoms where an absorption edge is accessible in the tender x‐ray energy range. A limitation of this approach is that it is not applicable to powder samples. For accurate determination of the absolute configuration requires correct identification of the exact Laue indices of each of the diffraction peaks. This cannot be achieved solely from the peak position, as reflections hkl and $\bar{h}\bar{k}\bar{l}$ occur at identical scattering angles and are therefore indistinguishable on this basis. Consequently, correct indexing of the diffraction data is essential to assign the correct reciprocal‐space coordinates. It should be noted that the present approach is applicable to enantiomorphic conglomerate crystals. More complex cases, including racemates, nonracemic twins, and solid solutions, do not permit a straightforward assignment of chirality using this approach alone and require complementary methods for the absolute configuration assignment [20]. Furthermore, prior knowledge of the crystal structure is required to calculate the corresponding structure factors and enable quantitative comparison with the experimental data.

A key advantage of the present approach is that the absolute configuration can be determined from a single Bragg reflection, without the need to measure the corresponding Friedel counterpart ($\bar{h}\bar{k}\bar{l}$). This is particularly beneficial in experimental techniques where access to Friedel pairs is limited or inaccessible, such as in grazing incidence x‐ray diffraction (GIXD). Although synchrotron radiation provides the most versatile platform for x‐ray energy dependent resonant scattering measurements, recent advances in laboratory based x‐ray absorption instrumentation have extended access to energy ranges near the absorption edges of various transition metals and heavier atoms [34]. Such developments may broaden the applicability of this method and reduce the dependence on synchrotron facilities in future studies. Overall, the single‐reflection method is a practical strategy for determining the absolute configuration, even where conventional approaches relying on multiple Friedel pairs are not applicable.

## 6 Conclusions

A key outcome of this study is that the absolute configuration of the Br‐oxo molecule with known crystal structure can be reliably determined from the x‐ray energy dependent resonant scattering behavior of a single Bragg reflection. The single reflection approach reduces the reliance on a statistically significant number of reflections sensitive to the absolute configuration compared with conventional SCXRD methods. The resulting x‐ray energy dependent variations in the resonant scattering signal provided a direct and unambiguous probe of the chirality. More broadly, this approach is applicable to molecular systems that are intrinsically chiral or form chiral crystals, provided that the absorption edge of at least one constituent atom is accessible. This method thus establishes an alternative pathway for absolute configuration determination, particularly for systems where only a limited number of suitable reflections are accessible.

## Funding

The authors thank the Belgian National Fund for Scientific Research (FNRS) for financial support through research projects: PICHIR PDR T.0094.22, DIFFRA GEQ U.G001.19, POLYP EQP U.N032.21F, POLYP2 EQP U.N03323F, CHIRI CDR J. 0088.24, CISSCA WEAVE T.W.023.23, 2Dto3D EOS No. 30489208, and CHISUB EOS No. 40007495. F.G. acknowledges funding from the Austrian Science Fund (FWF) and the project NEPHEWS under grant Agreement No. 101131414 from the EU Framework Program for Research and Innovation Horizon 2020 Europe.

## Supporting information




**Figure S1:** Comparison of the cross‐sectional intensity profiles of the 211 reflection measured below (13.43 keV in blue) and above (13.53 keV in orange) the Br K‐edge.
**Figure S2:** Calculated differences between structure factors of Friedel pairs on (A) an absolute scale and (B) a relative scale, averaged over the x‐ray energy range of 13.43–13.53 keV. The highlighted peaks include the 211 reflection, which shows a large relative intensity difference with respect to its Friedel pair; the 211 reflection, which exhibits a large absolute difference; and the 200 reflection, which shows no intensity difference between Friedel pairs.
**Figure S3:** Comparison of measured 211 and $\bar{2}\;\bar{4}\;\bar{2}$ Bragg peaks as a function of x‐ray energy with calculated structure factors for the (Ra) and (Sa) absolute configurations of the Br‐oxo. The x‐ray energy dependence of calculated values of the intensity of 211 and $\bar{2}\;\bar{4}\;\bar{2}$ reflections, averaged over all symmetry‐equivalent reflections. The red markers highlight measurements collected at the starting x‐ray energy of 13.43 keV after completion of the x‐ray energy dependent measurement series.
**Table S1:** Experimental and crystallographic parameters for Br‐oxo crystals before and after the x‐ray energy scan (13.43–13.53 keV).
**Figure S4:** Images of the Br‐oxo crystal recorded before and after the x‐ray energy dependent scan, showing a visible color change from colorless to brown, indicative of radiation damage.

## Data Availability

The data supporting the findings of this study are available within the article and its published Supporting Information, including details of the resonant scattering and SCXRD measurements, as well as the crystallographic data (PDF). The related data and codes are available from the corresponding repository https://doi.org/10.3217/4vcte‐sjb60.
