# Supplementary material for: Absolute Configuration Assignment From a Single Reflection Using Resonant X‐Ray Diffraction
Source: Chirality. 2026 Aug 4;38(8):e70129. doi: 10.1002/chir.70129 (PMC13434679; doi:10.1002/chir.70129)
Supplement: Supplementary file 1 [file CHIR-38-e70129-s001.docx]

Supporting information

Absolute configuration assignment from a single reflection using resonant X-ray diffraction

**Anmol Andotra^a,b^, Fabian Gasser^a^,** **Ferdinando Malagreca^b^, Roland C. Fischer^c^, Yves H. Geerts^b,d^, Roland Resel^a*^**

^a^ Institute of Solid-State Physics, Graz University of Technology, Petersgasse 16, 8010 Graz, Austria

^b^ Université Libre de Bruxelles (ULB), Faculté des Sciences, Laboratoire de Chimie des Polymères, CP 206/01, Boulevard du Triomphe, 1050 Bruxelles, Belgium

^c^ Institute of Inorganic Chemistry, Graz University of Technology, Stremayrgasse 9/V, A 8010 Graz, Austria

^d^ International Solvay Institutes of Physics and Chemistry, Université Libre de Bruxelles (ULB), CP 231, Boulevard du Triomphe, 1050 Bruxelles, Belgium

Table of Contents

[S1. Resonant Scattering	3](#_Toc227156656)

[S2. Conventional Single Crystal X-ray Diffraction (SCXRD)	5](#_Toc227156657)

S1. Resonant Scattering

Resonant scattering experiments were performed on the Br-oxo crystal. Fig. S1 shows a cross-sectional intensity profile of the 211 reflection extracted from the measured diffraction patterns below (13.43 keV) and above (13.53 keV) the Br K-edge. Above the absorption edge, the increase in background intensity arising from fluorescence emission from the crystal is accompanied by a reduction in the intensity of the 211 reflection. The X-ray energy dependence of the structure factor was calculated based on the experimentally determined atomic form factor of bromine atom in combination with the reported crystal structure of the Br-oxo ^1^. An overview of the intensity differences between Friedel pairs near the Br absorption edge is shown in Fig. S2 both on an absolute as well as relative scale. The intensities of the selected $211$, and $\bar{2}\bar{4}\bar{2}$ reflections were compared with calculated values and those extracted experimentally from data collected at X-ray energies ranging from 13.43 to 13.53 keV (Fig. S3).

Figure S1 Comparison of the cross-sectional intensity profiles of the $211$ reflection measured below (13.43 keV in blue) and above (13.53 keV in orange) the Br K-edge.

Figure S2 Calculated differences between structure factors of Friedel pairs on (A) an absolute scale and (B) a relative scale, averaged over the X-ray energy range of 13.43 keV to 13.53 keV. The highlighted peaks include the $211$ reflection, which shows a large relative intensity difference with respect to its Friedel pair; the $211$ reflection, which exhibits a large absolute difference; and the $200$ reflection, which shows no intensity difference between Friedel pairs.

Figure S3 Comparison of measured $211$ and $\bar{2}\bar{4}\bar{2}$ Bragg peaks as a function of X-ray energy with calculated structure factors for the (*R_a_*) and (*S_a_*) absolute configurations of the Br-oxo. The X-ray energy dependence of calculated values of the intensity of $211$ and $\bar{2}\bar{4}\bar{2}$ reflections, averaged over all symmetry-equivalent reflections. The red markers highlight measurements collected at the starting X-ray energy of 13.43 keV after completion of the X-ray energy dependent measurement series.
S2. Conventional Single Crystal X-ray Diffraction (SCXRD)

For SCXRD analysis, the complete data set collected at the starting X-ray energy of 13.43 keV was used. The diffraction data were indexed and integrated using XDS ^2^. The crystal structure was solved with Olex2 ^3^ using the SHELXT^4^ program via intrinsic phasing and subsequently refined with SHELXL ^5^ using full-matrix least-squares minimization. In the final refinement cycles, all non-hydrogen atoms were refined anisotropically.

The structure solution and refinement were carried out using same the Br-oxo crystal data collected at 13.43 keV. The refined Flack parameter converged to -0.055(6), close to zero to reliably establish the absolute configuration (Table S1, Supporting Information). The resulting assignment corresponds to the $R_{a}$ absolute configuration and agrees with the configuration determined using the X-ray energy dependent resonant scattering approach.

Table S1 Experimental and crystallographic parameters for Br-oxo crystals before and after the X-ray energy scan (13.43-13.53 keV).

| Molecule | Br-oxo (before X-ray energy scan) | Br-oxo (after X-ray energy scan) |
| --- | --- | --- |
| Formula | C_14_H_18_NO_2_Br | C_14_H_18_NO_2_Br |
| Crystal System | Orthorhombic | Orthorhombic |
| Space Group | *P*2_1_2_1_2_1_ | *P*2_1_2_1_2_1_ |
| Wavelength | 0.92320 | 0.92320 |
| Temperature (K) | 100.00 | 100.00 |
| $a (Å)$ | 7.5260(15) | 7.5840(15) |
| $b (Å)$ | 12.823(3) | 12.916(3) |
| $c (Å)$ | 15.038(3) | 14.994(3) |
| $\alpha(^{\circ})$ | 90 | 90 |
| $\beta(^{\circ})$ | 90 | 90 |
| $\gamma(^{\circ})$ | 90 | 90 |
| Volume (${Å}^{3}$) | 1451.3(5) | 1468.7(5) |
| $Z/Z^{'}$ | 4/1 | 4/1 |
| $F$ (000) | 619.768 | 615.766 |
| Flack parameter | -0.055(6) | -0.052(8) |
| GOF on $F^{2}$ | 1.0196 | 1.0621 |
| $R$ [on $F$, obs refs only] | 0.0305 | 0.0412 |
| $wR$ [on $F^{2}$, all data] | 0.0882 | 0.1245 |

At the beginning of the measurement, the Br-oxo crystal was colourless and transparent; however, after completion of the X-ray energy scan it appeared distinctly brown. The corresponding camera images (Figure S4) provide visual evidence of radiation damage arising during X-ray exposure. Despite this change, the Flack parameter and reliability factor (R) remained largely unchanged after the X-ray energy scan, with the Flack parameter refining to -0.055(6) and -0.052(8) and R to 0.0305 and 0.0412, respectively (Table S1).

Figure S4 Images of the Br-oxo crystal recorded before and after the X-ray energy dependent scan, showing a visible color change from colorless to brown, indicative of radiation damage.

**References**

1.	Andotra A, Das S, Killalea CE, Malagreca F, Zuffa C, Pandey P, et al. Isomorphism in a Series of Five Homologous Compounds. Crystal Growth & Design 2026 Apr 20. https://doi.org/10.1021/acs.cgd.6c00230

2.	Kabsch W. *XDS*. Acta Crystallogr D Biol Crystallogr. 2010 Feb 1;66(2):125–132. https://doi.org/10.1107/S0907444909047337

3.	Dolomanov O V., Bourhis LJ, Gildea RJ, Howard JAK, Puschmann H. OLEX2: A complete structure solution, refinement and analysis program. Journal of Applied Crystallography 2009;42(2):339–341. https://doi.org/10.1107/S0021889808042726

4.	Sheldrick GM. SHELXT - Integrated space-group and crystal-structure determination. Acta Crystallographica Section A 2015 Jan 1;71(1):3–8. https://doi.org/10.1107/S2053273314026370

5.	Sheldrick GM. Crystal structure refinement with SHELXL. Acta Crystallographica Section C Structural Chemistry 2015 Jan 1;71:3–8. https://doi.org/10.1107/S2053229614024218
